# Optimised Production and Extraction of Astaxanthin from the Yeast *Xanthophyllomyces dendrorhous*

**DOI:** 10.3390/microorganisms8030430

**Published:** 2020-03-19

**Authors:** Zuharlida Tuan Harith, Micael de Andrade Lima, Dimitris Charalampopoulos, Afroditi Chatzifragkou

**Affiliations:** 1 Faculty of Agro Based Industry, Universiti Malaysia Kelantan Jeli Campus, Jeli 17600, Kelantan, Malaysia; zuharlida84@gmail.com; 2 Department of Food and Nutritional Sciences, University of Reading, Whiteknights, P.O. Box 226, Reading RG6 6AP, UK; mid28@aber.ac.uk (M.d.A.L.); d.charalampopoulos@reading.ac.uk (D.C.); 3 Institute of Biological, Environmental and Rural Sciences (IBERS), Aberystwyth University, Gogerddan, Aberystwyth SY23 3EB, UK

**Keywords:** astaxanthin, *X. dendrorhous*, yeast cell, bioreactor, extraction, enzyme treatment, supercritical CO_2_, pigment

## Abstract

Currently, astaxanthin demand is fulfilled by chemical synthesis using petroleum-based feedstocks. As such, alternative pathways of natural astaxanthin production attracts much research interest. This study aimed at optimising bioreactor operation parameters for astaxanthin production and evaluating strategies for its subsequent extraction. The effect of pH and agitation was evident, as a significant reduction in both biomass and astaxanthin production was observed when the culture pH was not controlled and a low agitation speed was applied. At controlled pH conditions and a high agitation speed, a significant increase in biomass (16.4 g/L) and astaxanthin production (3.6 mg/L) was obtained. Enzymatic yeast cell lysis using two commercial enzymes (Accellerase 1500 and Glucanex) was optimised using the central composite design of experiment (DoE). Accellerase 1500 led to mild cell disruption and only 9% (*w/w*) astaxanthin extraction. However, Glucanex treatment resulted in complete astaxanthin extractability, compared to standard extraction method (DMSO/acetone). When supercritical CO_2_ was employed as an extraction solvent in Accellerase-pre-treated *Xanthophyllomyces dendrorhous* cells, astaxanthin extraction increased 2.5-fold. Overall, the study showed that extraction conditions can be tailored towards targeted pigments present in complex mixtures, such as in microbial cells.

## 1. Introduction

Astaxanthin has numerous applications in various sectors, including food, feed, and nutraceutical industries, as a pigment that possesses high antioxidant capacity [1]. In applications such as animal feed, whole microbial cells can be formulated as a feed ingredient without the need for pigment extraction. However, for more bespoke applications, such as cosmetic products and as food ingredients, pigment extraction is necessary to allow further purification processes to take place. Since microbial astaxanthin is produced intracellularly, efficient extraction strategies should allow the extracted pigment to retain its stability, colour, and bioactivity. The purity of extracted astaxanthin determines its market price, which may range from $2500 to $7000 per kg [2]. Natural astaxanthin is produced by microbial fermentation using either yeasts (such as *Xanthophyllomyces dendrorhous*) or microalgae (such as *Haematococcus pluvialis*). High astaxanthin yields and productivities that have been associated with strains of *X. dendrorhous*, render this microorganism a promising choice for the potential scaling up and commercialisation of astaxanthin production [3,4,5], while its extraction and purification still contributes to the overall complexity and cost of the whole production process.

Various cultivation modes, including batch, fed-batch, and continuous, have been investigated for carotenoid production in yeasts, either in the lab or at the pilot scale [6,7,8]. In the case of some yeast species cultivated on batch mode, high initial carbon concentrations (usually glucose) result in suppression of cell growth as well as product formation due to the Crabtree effect [9,10]. It has been demonstrated that *X. dendrorhous* undergoes the Crabtree effect, a phenomenon where cells metabolically switch to fermentative metabolism leading to ethanol production even under ample oxygen supply when initial glucose present is above a given threshold (strain dependent) [10,11]. Fed-batch cultivation mode is considered as an appropriate strategy to overcome such issues, as it allows the addition of one or more nutrients to the reactor during fermentation in order to maintain the concentration of the substrate below its inhibitory levels [12]. Furthermore, with regards to media optimization, low concentrations of ammonium and phosphate have been reported to be favourable towards astaxanthin production, whereas increased levels of citrate in the growth medium have been shown to stimulate astaxanthin production, as citrate can act as a carbon source towards astaxanthin biosynthesis in *Phaffia rhodozyma* [13]. The effect of light on astaxanthin productivity in several yeast and microalgae strains has been also extensively studied [14,15,16]. In the case of yeasts, Vázquez [17] and Stachowiak [18] reported enhanced astaxanthin volumetric production (ranging between 5%−15%) in *Phaffia rhodozyma* cultures subjected to illumination, in a strain-dependent manner. Notable increase in astaxanthin production has been reported in *X. dendrorhous* cultures, using ultraviolet and white light, reaching purities up to 77% due to suppression of β-carotene content [19]. Cell disruption is the first step in a downstream process for intracellular metabolites. A yeast cell wall is mainly composed of mannose polymers (±40% of dry cell mass) and β-glucans (±60% of dry cell mass) [20]. Various chemical methods act based on the permeability of a chemical agent inside the cell wall and allow periplasmic products to permeate through the yeast cell wall [21]. Several cell disruption methods have been investigated for astaxanthin extraction from yeast cells, including chemical, mechanical, and enzymatic methods, either solely or in combination. Mechanical methods that have been studied for the extraction of carotenoids, enzymes, and lipids from several yeast cells, primarily at a small scale, include homogenisation and utilisation of glass beads [22,23,24] or ultrasonication waves [21]. The disadvantage of the mechanical disruption methods is that they are not product selective, as a variety of cellular components are also released along with astaxanthin [21]. Enzymatic cell lysis has been extensively applied for the production of yeast extract, mainly from strains of *Saccharomyces cerevisiae* [25]. However, only a few research studies have focused on astaxanthin extraction from *X. dendrorhous* using an enzymatic cell lysis approach. The complex structure of yeast cells requires the synergistic effect of several enzyme activities, including protease and β-glucanase, in order to disrupt the outer layer of their cell wall [26] prior to astaxanthin extraction. The selection of suitable enzymes is critical to ensure a high degree of cell wall disruption that subsequently results in high astaxanthin extractability. This method is considered a much gentler process than mechanical or chemical disruption strategies. However, the enzymatic method might be considered as less cost-effective due to the potentially high cost of the enzymes [21].

In addition to the above methods, supercritical fluid extraction has been applied for the extraction of carotenoids from various sources, including plants, microalgae, and yeast. This method is considered a “green” technology and has the potential to replace the commonly used solvent-based extraction methods for the selective recovery of carotenoids. Furthermore, the low critical temperature of CO_2_ means that this process could be carried out at moderate temperatures, thus preventing the degradation of carotenoids, which can occur due to their sensitivity to relatively high temperatures [27]. Supercritical CO_2_ has been successfully used to extract compounds such as lipids, phenolic compounds, and pigments from plants, yeast, and bacteria. However, very few research studies have been conducted on astaxanthin extraction from *X. dendrorhous*.

This study aimed to investigate the production of astaxanthin in *Xanthophyllomyces dendrorhous* DSMZ 5626, using semi-defined media to optimise the fermentation parameters in lab-scale bioreactor cultures. Then, several methods for astaxanthin extraction were assessed, including enzymatic lysis and supercritical CO_2_. A design of experiment (DoE) approach was used to identify the optimal process conditions for astaxanthin extraction in the case of the enzymatic lysis and supercritical CO_2_ methods and generate new knowledge on the effect of process conditions that can form the basis for developing a scalable and efficient strategy for microbial astaxanthin extraction.

## 2. Materials and Methods

### 2.1. Microorganism and Culture Media

The yeast strain *Xanthophyllomyces dendrorhous* DSMZ 5626 was used in this study and was purchased from Leibniz Institute DSMZ. The strain was proliferated and maintained in Yeast and Mold (YM) media containing (in g/L): yeast extract (3.0); malt extract (3.0); peptone from soybean (5.0); glucose (10.0); and agar (15). Stock yeast cultures were stored at −80 °C until further use. 

For the preparation of the inoculum, a loopful of stock yeast culture was inoculated on sterilised commercial YM agar (Sigma Aldrich, Dorset, UK) and incubated at 20 °C for 5 days. After that, a single colony of yeast *X. dendrorhous* was transferred in 30 mL of YM broth media (similar composition as above) for cell proliferation and was incubated for 3 days prior to inoculation in semi-defined media. Finally, a suspension of *X. dendrorhous* was transferred into 50 mL of semi-defined media to a final optical density (OD) measurement of 0.1. The optical density measurement was conducted using a Biomate 3 UV/VIS Spectrophotomer (Thermo Electron Corporation, Massachusetts, US). The composition of the semi-defined media was as follows (in g/L): carbon source (30), yeast extract (2.0), malt extract (2.0), KH_2_PO_4_ (7.0), (NH_4_)_2_SO_4_ (1.0), MgSO_4_.7H_2_O (1.5), FeCl_3_.6H_2_0 (0.15), ZnSO_4_.7H_2_O (0.02), MnSO_4_.H2O (0.06), and CaCl_2_.2H_2_O (0.15). 

### 2.2. Bioreactor Fermentations

A 2-L stirred tank bioreactor (BIOSTAT B, Sartorious AG, Germany) was used with a working volume of 1.5 L. This unit consisted of a double jacketed 2-L vessel with an integrated digital control unit (DCU) that controlled the speed, air flow, temperature, and pH of the fermentation. The vessel was equipped with baffles, three units of six-bladed impellers, as well as multiple ports for sensors for fermentation control. The temperature was controlled by circulating warm/cold water through the double jacket vessel, whereas air was supplied via compressed air. The pH of the fermentation was controlled by an pH probe (Easy Ferm, Hamilton, Switzerland) and the pH corrective solutions used were 5 M sodium hydroxide and 5 M hydrochloric acid. Antifoam 204 (10% *v/v*) (Sigma Aldrich, Dorset, UK) was used to prevent foaming during fermentation. Dissolved oxygen (DO) was controlled by a DO-probe (OxyFerm, Hamilton, Switzerland). A total of 1.5 L of synthetic media was prepared, added in the reactor, and autoclaved at 121 °C for 20 min prior to yeast inoculation. Batch fermentation was carried out at different process parameters to investigate the best conditions for microbial astaxanthin production in *X. dendrorhous*, including (i) pH (pH 6 and uncontrolled pH) and (ii) agitation speed (250, 400, and 600 rpm). Other processing parameters were maintained as follows: temperature, 20 °C; aeration, 1 L/min. Fed-batch cultivation was also conducted using semi-defined media as described previously. Two different refined carbon sources, glucose and glycerol, were used as feeding solutions. 10 g/L of feed solution were added to the bioreactor once the values of dissolved oxygen increased during fermentation (DO-stat approach). Fermentation conditions were controlled as follows: temperature, 20 °C; pH 6; agitation speed, 600 rpm; aeration, 1 L/min. Samples were taken at regular time intervals for 5 days and analysed as described below.

### 2.3. Yeast Cell Preparation for Extraction

Prior to cell lysis experiments, 2 mL of fresh *X. dendrorhous* were added to 2 mL vials and centrifuged at 10,845× *g* for 10 min (Multifuge X3R, Fisher Scientific, Loughborough, UK). The pellets (~30 mg of cell pellet) were collected and washed twice using distilled water. In the case of wet cells extraction, fresh cells were kept at 4 °C until further use. To prepare dried cell samples, the vials containing wet cells were frozen at −20 °C and then freeze dried for 24 h (Virtis SP Scientific, Ipswich, UK); these pellet samples corresponded to about 30 mg of dry cells. 

In the case of astaxanthin extraction by supercritical CO_2_, 1.5 L of yeast culture medium was collected after 5 days of bioreactor cultivation and centrifuged at 10,845× *g* for 10 min (Multifuge X3R, Fisher Scientific, Loughborough, UK). The pellet was washed twice with distilled water, frozen at −20 °C for 24 h, and then freeze dried for 72 h (Virtis SP Scientific, Ipswich, UK) prior to SCFE-CO_2_ extraction. 

### 2.4. Enzymatic Cell Lysis and Astaxanthin Extraction

The enzymatic lysis of *X. dendrorhous* yeast cells was carried out using two commercial enzymes, Accellerase 1500 (DuPont, Stevenage, UK) and Glucanex (Novozyme, Bagsværd, Denmark). Accellerase 1500 contained multiple enzymatic activities, including cellulase, hemicellulase, and β-glucanase, whereas Glucanex contained β-1, 3 glucanase activity from *Trichoderma harzianum*. 

In order to measure the β-glucanase activity of the two enzymes used in this study and inform on the optimum enzyme dosage, the following method was used: 0.25 mL of enzyme solution and 0.25 mL of laminarin solution 1% (*w/v*) obtained from the seaweed *Laminaria digitata* (Sigma-Aldrich, Dorset, UK) in sodium acetate buffer (0.1 M, pH 5.5) were mixed and incubated at 55 °C for 30 min. The reaction was stopped by heating at 100 °C for 5 min and reducing sugars were determined by the method of 3, 5-dinitrosalicylic acid (DNS method) using glucose as the standard. For the control, distilled water was used instead of laminarin solution. One activity unit of β-1, 3 glucanase (U) was defined as the release of 1 µmol of glucose per min per mL of enzyme solution at 55 °C.

In order to determine the optimum conditions for enzymatic hydrolysis a non-factorial 2^2^ central composite design of experiments (DoE), with two factors at three levels, was employed. The two independent variables assessed were temperature and pH, with values reflecting their optimal activity conditions, as recommended by the manufacturer. The dependent variable was astaxanthin extractability (%, *w/w*), calculated by comparing the astaxanthin concentration obtained after each extraction method to the concentration obtained using the standard chemical extraction method by Sedmak et al. [28]; the method is described in detail below. Thirteen different experiments were carried out in total for each enzyme, which included the low, high, and axial points for all the parameters, along with a central point replicated five times to calculate experimental errors. Minitab 17 software was used for the experimental design and statistical analysis.

In the case of Accellerase 1500, 0.75 mL of sodium citrate buffer (pH varied according to the DoE) and 0.25 mL of enzyme were added to vials containing wet and freeze-dried cell biomass of *X. dendrorhous*. The mixture was then incubated in a thermomixer (Eppendorf, Stevenage, UK) under agitation at 1400 rpm for 1 h. After enzyme treatment, samples were centrifuged at 10,845× *g* for 10 min and the supernatant was discarded. The pellet was washed twice with distilled water to remove excess enzymes and buffer. Subsequently, 1 mL of acetone was added to the pellet and the suspension mixed at 2000 rpm for 10 min to facilitate astaxanthin recovery. A total of 100 µL of NaCl (20% *w/v*) was then added to the mixture to assist in the formation of the aqueous and organic (solvent) phases. Samples were mixed for another 5 min and were centrifuged at 5423× *g* for 5 min. The solvent phase (upper layer) was used for astaxanthin analysis. In the case of Glucanex treatment, 0.75 mL of sodium citrate buffer (pH varied according to the DoE) and 0.25 mL of enzyme (5% *w/v* of Glucanex stock solution) were added to vials containing wet and freeze-dried cell biomass of *X. dendrorhous*. Subsequent steps of enzyme incubation and recovery of astaxanthin were the same as in the case of the Accellerase 1500 treatment.

### 2.5. Supercritical CO_2_ Extraction of Astaxanthin

Freeze dried samples of either intact or enzymatically pre-treated *X. dendrorhous* cells were subjected to supercritical CO_2_ extraction in a rig (SciMed, Stockport, UK). The apparatus consisted of a recirculating chiller, a CO_2_ line, solvent and co-solvent pumps, a heat exchanger, a 200 mL extraction vessel, an automated backpressure vessel, a collection vessel, and a controller. For every run, 2.0 g of freeze-dried samples and 100 g of inert glass beads (5 mm) (Sigma Aldrich, Dorset, UK) were added to the extraction vessel and submitted to a CO_2_ flow rate of 15 g/min. 

In order to optimise the extraction process, a non-factorial 2^3^ central composite design of experiments (DoE) method with three factors and three levels was employed. The independent variables were temperature (50, 60, and 70 °C), co-solvent concentration (EtOH at 5%, 10%, and 15%, *v/v*) and pressure (150, 250, and 350 bar); each run lasted 30 min. The dependent variable accessed was astaxanthin extractability (%, *w/w*). Fourteen experiments, which included the low, high, and axial points of all the parameters, were conducted along with a central point, which was replicated three times. At the end of each run, astaxanthin was obtained in ethanol; the sample was immediately analysed for astaxanthin content using a spectrophotometer as described below. 

Response Surface Methodology (RSM) was used to construct a mathematical model describing the effectiveness of the astaxanthin extraction. All the independent variables of the model equation were tested statistically by the F-test at a 95% interval of confidence. The Coefficient of Variance (CV, %) and the Determination Coefficient (R^2^) were used to evaluate the quality of the fitted polynomial model. Finally, the experiments were validated by performing additional experiments (in triplicate) using the optimal conditions for astaxanthin extraction, as suggested by the model. The experimental values from these runs were then compared to the predicted values given by the model to confirm the accuracy of the model.

### 2.6. Analytical Methods

Astaxanthin concentration was determined by the following the method of Sedmak et al. [28]. Briefly, 1 mL of dimethyl sulfoxide (DMSO) was preheated at 55 °C and added to the freeze-dried biomass, followed by vortexing for 30–40 s. Subsequently, 0.1 mL of 20% sodium chloride (NaCl) and 1.0 mL of acetone were added to the mixture to extract the intracellular carotenoids. The aqueous and organic phase was separated by centrifugation at 5423× *g* for 5 min. The extraction process was repeated until a colourless biomass was obtained, the organic phases were pooled together, and their absorbance was measured at 480 nm in a Biomate 3 UV/VIS Spectrophotomer (Thermo Electron Corporation, Massachusetts, US). The resultant values were then divided by the extinction coefficient of 2150. This spectrophotometric method was also used to determine astaxanthin concentration following extraction with enzymatic cell lysis and supercritical CO_2_. The equation for the estimation of the total carotenoids’ concentration is given below:

(1) $$Carotenoids\;content\;(\mu g/g)\;=\;\frac{A\times V\;\left(mL\right)\times 10^{4}}{A_{1cm}^{1\%}\times P\;\left(g\right)}$$

where *A* is the absorbance at 480 nm, *V* is the volume, $A_{1cm}^{1\%}$ is the coefficient (2150), and *P* is the weight of biomass. 

Astaxanthin extractability (%) was calculated by the following equation (Machado et al., 2016).

(2) $$Astaxanthin\;extractability\;\left(\%\right)=\frac{C}{C_{DMSO}}\times 100$$

where *C* is the concentration of astaxanthin extracted from the cells using different extraction techniques and *C_DMSO_* is the total carotenoids extracted from *X. dendrorhous* using cell disruption with DMSO/acetone.

In the case of batch bioreactor cultures using semi-defined media, samples of ~2 mL were periodically withdrawn from the bioreactor. A total of 1 mL of the sample was added into a pre-dried tube (24 h in drying oven at 100 °C) and centrifuged at 10,845× *g* for 10 min. The supernatant was collected for HPLC analysis. The pellet was washed twice using distilled water and was frozen at −20 °C prior to freeze drying for 2 days (Virtis, Ipswich, UK). The dry weight of yeast biomass was calculated as the difference between the weight of the tubes before and after freeze drying process. Sugar substrate (glucose) and metabolites concentration (organic acids, ethanol, and glycerol) were analysed by high performance liquid chromatography (HPLC) using an Agilent Infinity 1260 system (Agilent Technologies UK Limited, Cheadle, UK) with an Aminex HPX-87H column (Bio-rad, Watford, UK) column coupled to a differential refractometer and a DAD detector. Operating conditions were as follows: sample volume: 20 µL; mobile phase: 0.5 mM H_2_SO_4_; flow rate: 0.6 mL/min; column temperature: 65 °C. The quantification of each chromatogram peak was achieved on the basis of external standard curves, using standard solutions of known concentrations for each compound.

### 2.7. Scanning Electron Microscopy

The morphology of the cell biomass samples after each treatment was analysed using a Quanta FEG 600 Environmental Scanning Electron Microscopy instrument (FEI Co. Inc., Hillsboro, Oregon). Samples were mounted onto SEM stubs using carbon tape and then sputter coated with a thin layer of gold to prevent charging during imaging. The parameters used for imaging were 20 kV of accelerating voltages, a 4.0 spot size, and a working distance of approximately 10–12 mm. Images were recorded under vacuum at 6000× magnification.

## 3. Results and Discussion 

### 3.1. Batch Bioreactor Fermentations Using Semi-Defined Media

**Figure 1:**
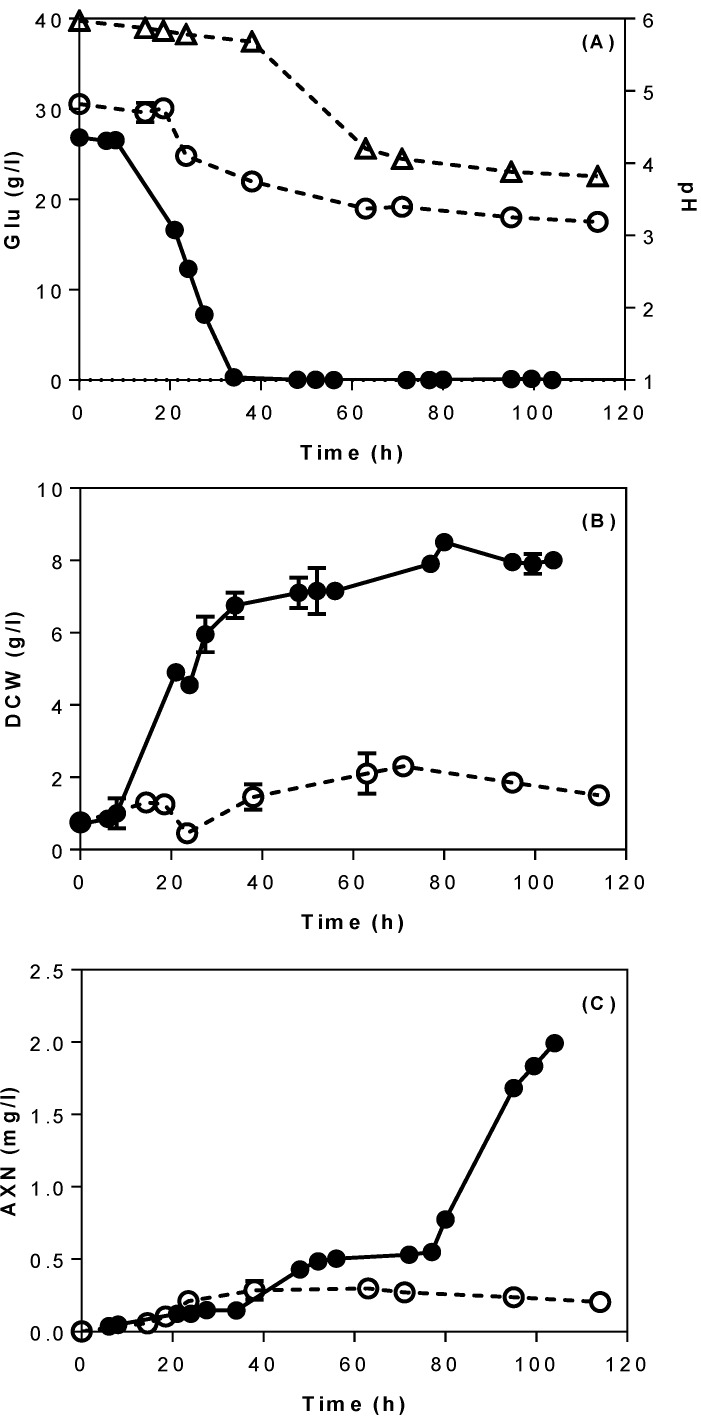
Profile of (**A**) glucose consumption, (**B**) growth and (**C**) astaxantin production during cultivation of *X. dendrorhous* in a 2-L stirred tank reactor using semi-defined media at different pH conditions. Symbols represent: (●) controlled pH 6, (○) uncontrolled pH, and (∆) pH profile for the uncontrolled experiment. Fermentation conditions; aeration, 1 L/min; temperature, 20 °C; agitation, 250 rpm. Abbreviations: AXN—astaxanthin; Glu—glucose; DWC—dry cell weight.

Initially, cultures of *X. dendrorhous* were performed in a 2-L bioreactor using semi-defined media with glucose (30 g/L) as carbon source, with a view to optimise the process parameters (pH and agitation) of the bioreactor operation. Figure 1 depicts the fermentation profile of *X. dendrorhous* DSMZ 5626 using semi-defined media in a 2-L stirred tank bioreactor. Around 8 g/L of dried biomass and 2 mg/L of astaxanthin were produced after 105 h under controlled pH conditions (Figure 1B,C). In the case of uncontrolled pH, the initial pH of the culture was 6.1, and was progressively decreased to 4.2 after 48 h of fermentation, at a time point in which cell growth was suppressed, as demonstrated by poor biomass (1.5 g/L) and astaxanthin production (0.2 mg/L). At the end of fermentation, the pH of the culture had dropped to 3.8, indicating a highly acidic culture environment due to the presence of acetic acid (~3 g/L). A low pH environment is known to cause acid stress in yeasts, resulting in reduced membrane permeability, anion extrusion, and alters expression of genes that are key for yeast growth [29,30]. 

As observed in Figure 1A, glucose consumption was strongly influenced by the pH. In uncontrolled pH cultures, around 10 g/L of glucose were consumed by the yeast before cell growth was interrupted at 36 h due to acidic conditions (pH 4.2). This is in contrast with yeast growth in controlled pH cultures, where 30 g/L of glucose were fully consumed within 34 h of fermentation. Additionally, yeast metabolism by-products such as ethanol (2 g/L) and glycerol (1.2 g/L) were produced during fermentation. Hu et al. [31] reported that the optimal pH for *X. dendrorhous* growth is at 6. When the pH largely deviates from its optimal range, it prevents the cells from maintaining their optimal intracellular pH, which in turn results in failure of key intracellular enzymatic functions [32]. 

**Figure 2:**
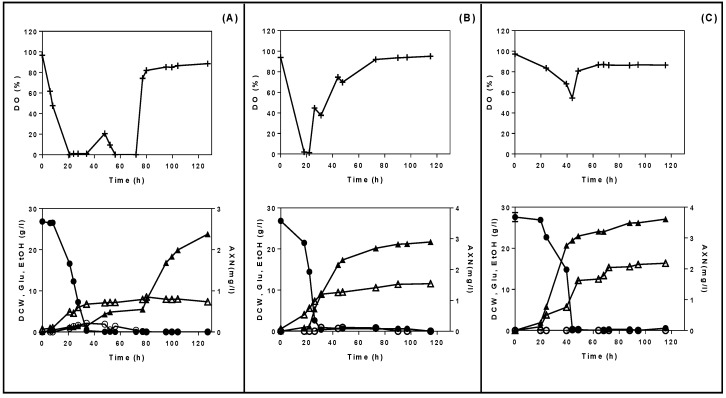
Effect of agitation speed on cell growth, glucose consumption, and astaxanthin and ethanol production in a 2-L bioreactor using semi-defined media with 30 g/L of glucose as carbon source. (**A**) 250 rpm; (**B**) 400 rpm; (**C**) 600 rpm. Fermentation conditions: aeration, 1 L/min; temperature, 20 °C; pH 6. Symbols represent: (▲) astaxanthin, AXN (mg/L); (●) glucose, Glu (g/L); (∆) dry cell weight, DCW (g/L); (○) ethanol, EtOH (g/L); (+) dissolved oxygen, DO (%).

Furthermore, the effect of agitation speed (250, 400, and 600 rpm) on astaxanthin production was investigated under a controlled pH (pH 6) and constant air flow rate (1 L/min). Figure 2 depicts the fermentation profile of *X. dendrorhous* in terms of yeast growth, astaxanthin production, dissolved oxygen, glucose consumption, and ethanol production, in cultures with semi-defined media and at different agitation speeds. It was clear that astaxanthin production was positively correlated with increasing agitation speed. Specifically, the highest agitation speed (600 rpm) supported both the highest astaxanthin yield (3.61 mg/L) and biomass production (16.35 g/L of dry weight) at 118 h of fermentation (Figure 2C). In contrast at the lowest agitation speed (250 rpm), 2.4 mg/L of astaxanthin were produced with 8 g/L of biomass. As for yeast growth, *X. dendrorhous* entered the stationary phase after 36 h of fermentation when 250 and 400 rpm were applied, due to oxygen limitations in the bioreactor (<20%) (Figure 2A,B). On the other hand, at high agitation (600 rpm), the cell growth had a longer exponential phase, up to 70 h of fermentation. 

Observing the dissolved oxygen values when agitation at 250 rpm was applied, DO readings fell at 0% after 20 h of fermentation and were maintained at low levels (<20%, *v/v*) until 72 h. After that, DO levels increased to >80%, coinciding with the stationary growth phase of *X. dendrorhous*. However, when the agitation rate was increased to 400 and 600 rpm, the DO levels remained at >40% for most of the fermentation duration. High agitation rates ensured sufficient dissolved oxygen supply that is required for cell growth. In terms of metabolites production, maximum ethanol concentration (EtOH_max_) was observed at 2.1 g/L after 31 h of fermentation when agitation at 250 rpm was applied. However, at agitation speeds of 400 and 600 rpm, minimal amounts of ethanol were detected in the culture (<1 g/L). This reduction in ethanol production indicated that the Crabtree effect was reduced at higher agitation rates. It should be noted that in all experiments, any produced ethanol was subsequently consumed by the yeast once glucose was depleted from the media. High agitation rates ensure a better oxygen uptake rate by microorganisms as well as heat and mass transfers, enabling a satisfactory supply of nutrients and facilitating the removal of carbon dioxide from the culture medium [33]. It has been reported that medium to high agitation rates (300–900 rpm) increased yeast growth and astaxanthin production in *X. dendrorhous* [34,35]. Sufficient supply of oxygen can enhance astaxanthin production by preventing NADH accumulation and supplying oxygen molecules to re-oxidize NADH into NAD+ as a starting material for astaxanthin biosynthesis [10]. 

Yamane et al. [10] investigated the effect of oxygen supply on growth and astaxanthin production of *X. dendrorhous* by controlling the DO levels in the fermenter. They found out that increasing the DO (from 20% to 80%) benefited both biomass and astaxanthin production and could inhibit the occurrence of the Pasteur and Crabtree effects, which are related to high glucose concentrations. The Pasteur effect refers to the inhibition of glycolysis by respiration whereas the Crabtree effect refers to the inhibition of respiration by glycolysis [10,36]. 

### 3.2. Fed-Batch Bioreactor Fermentations Using Semi-Defined Media

**Figure 3:**
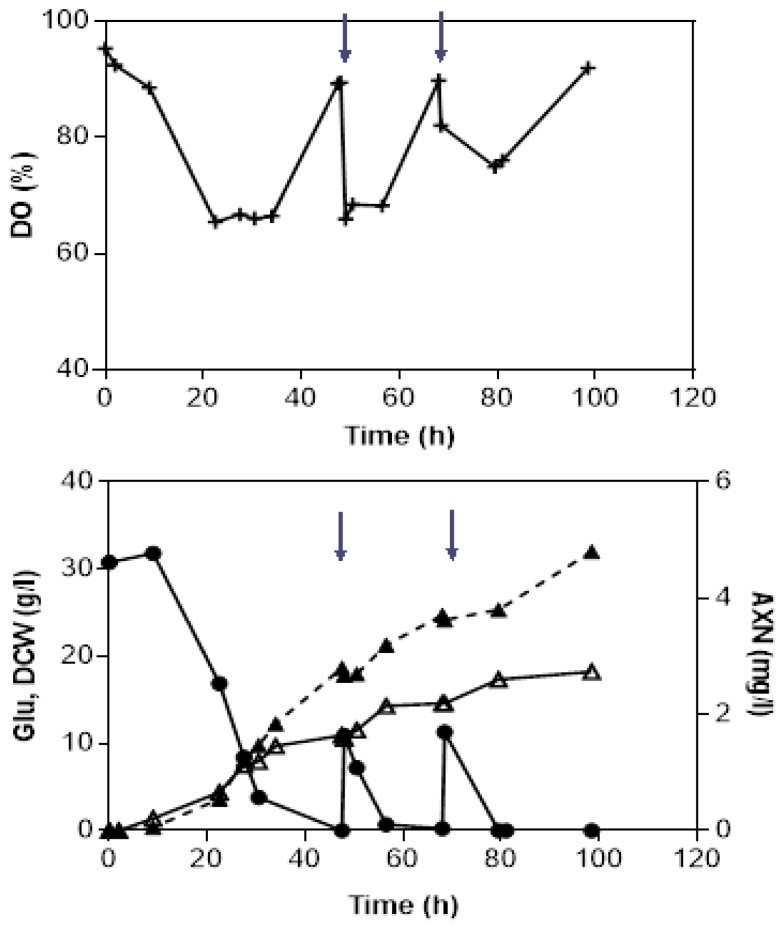
Fed-batch cultivation of *X. dendrorhous* in a 2-L stirred tank bioreactor. Fermentation conditions: aeration, 1 L/min; temperature, 20 °C; pH 6. Symbols represent: (▲) astaxanthin, AXN (mg/L); (●) glucose, Glu (g/L); (∆) dry cell weight, DCW (g/L); (○) ethanol, EtOH (g/L); (+) dissolved oxygen, DO (%). Arrows indicate feeding points.

A key-objective of this study was to investigate the feasibility of the fed-batch strategy on the improvement of biomass and astaxanthin yield in *X. dendrorhous*. To this end, dissolved oxygen control (DO-stat) was used as a substrate feeding indicator, due to the reverse dependence of a carbon source with dissolved oxygen in the culture. In batch fermentations, a sharp increase in dissolved oxygen levels was observed, demonstrating a slow oxygen uptake by the microorganism, as a response to the starvation of nutrients in the fermenter. That was the point of the feeding intervention applied in fed-batch cultivations. Figure 3 depicts the fed-batch fermentation kinetics for *X. dendrorhous* cultivated in semi-defined media with glucose as feeding solution. Glucose was added at two time points (48 and 68 h) where DO values spiked from <68% to 89%, coinciding with the depletion of glucose in the fermenter (residual glucose concentration, 0–1 g/L). Following feeding of glucose, DO values reduced again, indicating cell activity. After 100 h of fermentation, 18 g/L of biomass and 4.8 mg/L of astaxanthin were observed after two feeding cycles. Overall, astaxanthin production increased by 33% compared to the respective fermentation with glucose in batch mode (3.6 mg/L).

Fed-batch strategy has been implemented before to improve both yeast production and astaxanthin pigmentation in *X. dendrorhous*. Liu and Wu [37] studied various feeding schemes including constant, exponential, and optimal feeding (based on a mathematical model) with glucose as carbon source. They found out that the optimal feeding scheme resulted in high biomass (29 g/L) and astaxanthin production (27 mg/L) compared to batch mode (16.8 g/L of biomass and 15 mg/L of astaxanthin). Besides that, the fed batch approach has been widely implemented as a strategy to improve biomass and astaxanthin production using low cost substrates as carbon sources, such as molasses, wood hydrolysates, and date juice in *X. dendrorhous* [34,38,39]

### 3.3. Enzymatic Cell Disruption and Astaxanthin Extraction 

Wet and dried *X. dendrorhous* cells were subjected to enzymatic treatment using Accellerase 1500, aiming to disrupt the cell wall. Accellerase 1500 is an enzyme cocktail that has multiple enzymatic activities, including cellulases and hemicellulases, and a low measured β-glucanase activity of 0.03 U/mL. A full factorial central composite design of experiments (CCD) was employed to identify the optimal conditions for yeast cell disruption. The parameters that were investigated were temperature (30, 40, and 50 °C) and pH (4.5, 5.5, and 6.5). In total, thirteen runs, including five zero points for error estimation, were conducted, and the corresponding results are presented in Table 1. 

Overall, the use of wet cells resulted in higher astaxanthin extractability compared to freeze dried cells. A possible explanation for this might be due to the presence of moisture inside the cells, which resulted in a higher water activity and potentially a higher enzymatic activity towards *X. dendrorhous;* such an effect of water activity on enzyme reaction has been reviewed by Rezaei et al. [40]. On the other hand, in the freeze-dried samples, the moisture levels were much lower due to the drying process. Amongst the dried samples, the highest astaxanthin extractability was observed for Run 5 (temperature at 40 °C, pH 4.09), whilst the lowest extractability was observed for Run 8 (temperature at 54.1 °C, pH 5.51). 

The results obtained from the thirteen runs were used to construct two quadratic models, one for dry cells (Equation (3)) and one for wet cells (Equation (4)), describing the main, interaction, and quadratic effects of the independent variables (pH, temperature) on the response variable (astaxanthin extractability).

(3) $$Astaxanthin\;\;Extractability\;\left(\%\right)\;=\;-44.1+8.29pH+1.679T-0.958pH^{2}-0.02412T^{2}+0.0339pH.T$$

(4) $$Astaxanthin\;\;Extractability\;\left(\%\right)\;=\;-73.7+16.43pH+2.544T-1.714pH^{2}-0.03362T^{2}+0.0016pH.T$$

where *T* = temperature (°C) of extraction and astaxanthin extractability (%) = % *w/w* astaxanthin extracted from yeast cells.

Even though astaxanthin extractability was higher using wet cells as compared to dry ones, the models generated for both samples demonstrate similar profiles in terms of the significance of the variables tested. With regards to astaxanthin extractability using dry cells, both linear and quadratic terms had a statistically significant (*p* < 0.05) effect on the extraction. More specifically, both linear terms of pH and temperature had a negative correlation with astaxanthin extractability, where an increase in temperature and pH resulted in decreased astaxanthin extractability (%). However, the interactions of the linear terms (pH*T) showed no significant effect (*p* > 0.05). Similar results were observed with fresh cells. In order to confirm the accuracy of the model, an analysis of variance (ANOVA) was performed along with an F-test for validation. In terms of the astaxanthin extractability using freeze dried cells, the fit of model, expressed by the coefficient of regression R^2^ value, was 0.909. Furthermore, the F-value (14.09) obtained was higher than the tabulated F-value (F_5,7_ = 3.97). In terms of the wet cells, the regression R^2^-value, was 0.9631, whereas the F-value (36.57) was also higher than the tabulated F-value (F_5,7_ = 3.97). Hence, it can be deduced that the models for both freeze dried and fresh samples can satisfactorily describe the extraction process.

**Figure 4:**
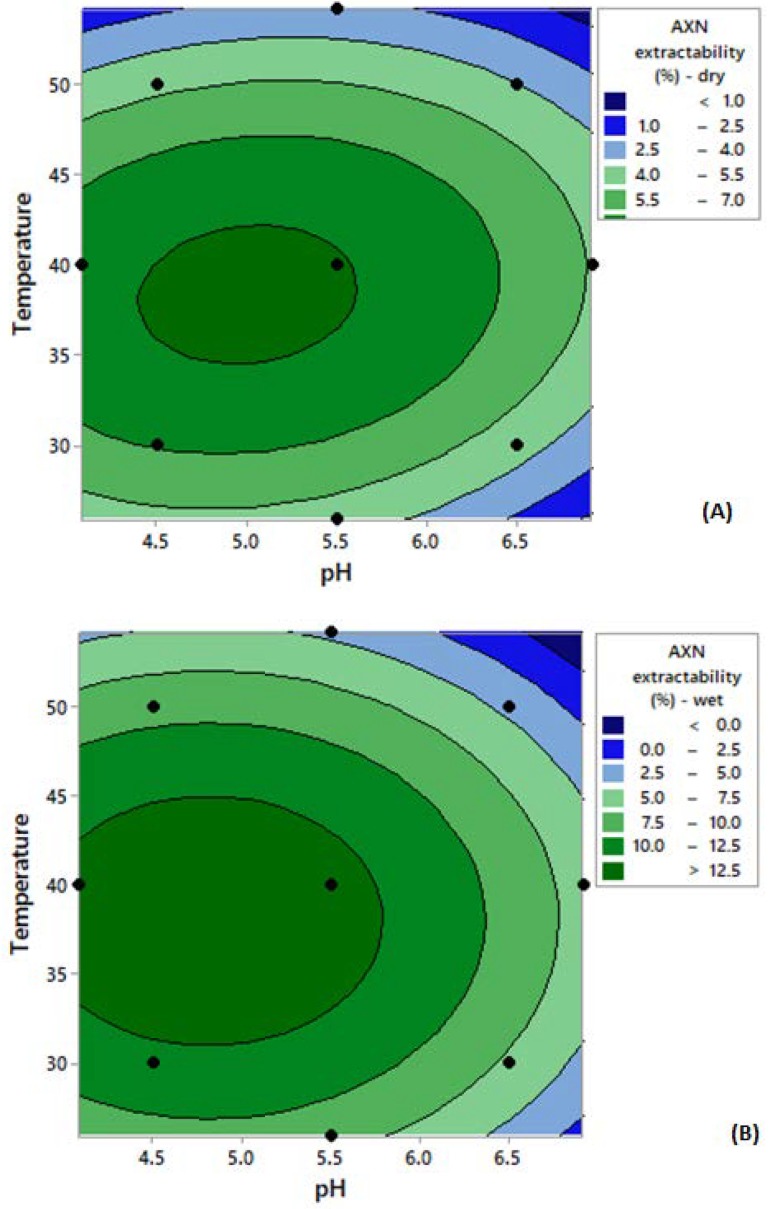
Contour plots depicting the effect of temperature and pH on astaxanthin extractability from *X. dendrorhous* cells following Accellerase 1500 treatment on (**A**) dry cells and (**B**) wet cells. * The black dots on the contour plot represent the experimental points. Abbreviation: AXN—astaxanthin.

Figure 4 depicts the two-dimensional contour surface graphs generated, which provide the model prediction for astaxanthin extractability using Accellerase 1500. For both wet and dried cells, higher astaxanthin extractability (around 9%–14%, *w/w*) was obtained at an extraction temperature around 35–40 °C and a pH around 4.5–5.5. Based on the model prediction, the optimum astaxanthin extractability for both samples could be obtained at a temperature of 38.8 °C and pH of 4.97. This set of values predicted an astaxanthin extractability of 8.85% (dry cells) and 14.09% (wet cells), respectively. The models were subsequently validated by repeating the experiments at these critical parameters and astaxanthin values were similar, although slightly higher than the predicted ones (10.25% *w/w* for dry cells and 14.99% *w/w* for wet cells). These results demonstrated that the generated models can be considered valid to describe the extraction process.

The Glucanex enzyme was also investigated in this study, as a means of yeast cell disruption. Glucanex is a lytic enzyme from *Trichoderma harzianum* with a measured β-glucanase activity of 0.4 U/mL. A response surface design was also used in this study with two variables being investigated (temperature and pH). A full factorial CCD was applied for this purpose. The parameters that were investigated were temperature (35, 45, and 55 °C) and pH (3.5, 4.5, and 5.5). In total, thirteen factorial runs, including five zero points for error estimation, were conducted and the results are presented in Table 2. Similar to observations with Accellerase 1500, Glucanex treatment on wet cells resulted in significantly higher astaxanthin extraction compared to dry cells. However, overall astaxanthin extractability was much higher in the case of Glucanex than Accellerase 1500. The highest astaxanthin extractability was observed at Run 5 (temperature at 30.9 °C and pH 4.5) (115%) whilst the lowest extractability was observed at Run 4 (temperature at 55 °C and pH 5.5) (43%). Overall, the results indicated that both pH and temperature play a critical role in determining the extraction of astaxanthin by *X. dendrorhous* using Glucanex. 

The results obtained from the thirteen runs with Glucanex enzyme were used to construct two quadratic models (Equation (5) for dry cells and Equation (6) for wet cells), describing the main, interaction, and quadratic effects of the independent variables (pH and temperature) on the response (astaxanthin extractability).

(5) $$Astaxanthin\;Extractability\;\left(\%\right)\;=\;-201-0.20T+143.1pH-0.0241T^{2}-15.59pH^{2}-0.026pH.T$$

(6) $$Astaxanthin\;Extractability\;\left(\%\right)\;=\;-275.1+170pH+1.35T-18.71pH^{2}-0.0442T^{2}+0.038pH.T$$

where *T* = temperature (°C) and astaxanthin extractability (%) = % (*w/w*) astaxanthin extracted from the cells.

To confirm the accuracy of the models, an analysis of variance (ANOVA) was performed along with an F-test for validation. In the case of the freeze-dried cells, the coefficient of regression R^2^-value was 0.9492, whereas the F-value (26.17) obtained was higher than the tabulated F-value (F_5,7_ = 3.97), indicating the good fit of the model. It was observed that only the linear term of temperature and the quadratic term of pH had a statistically significant (*p* < 0.05) influence on the extraction, whereas the linear term of temperature, the quadratic term of pH, and the interaction between pH and temperature did not have a significant effect (*p* > 0.05). In the case of the wet cells, the coefficient of regression R^2^-value was 0.9870, whereas the F-value (106.11) was also higher than the tabulated F-value (F_5,7_ = 3.97), indicating the good fit of the model. Similar to the dried cells, the interaction between pH and temperature did not have a significant effect (*p* > 0.05) on astaxanthin extractability.

**Figure 5:**
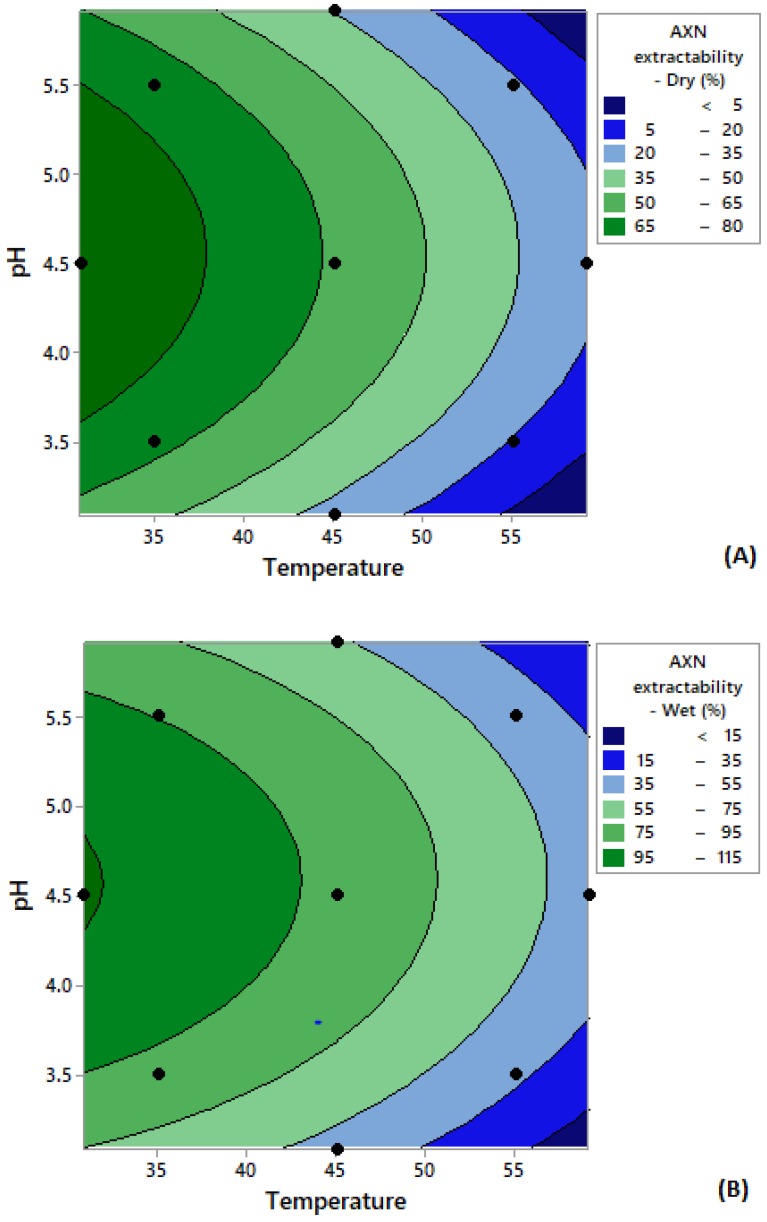
Contour plots depicting the effect of temperature and pH on astaxanthin extractability from *X. dendrorhous* cells following Glucanex on (**A**) dry cells and (**B**) wet cells. * The black dots on the contour plot represent the experimental points. Abbreviation: AXN—astaxanthin.

The model prediction is presented in the two-dimensional contour surface graphs generated (Figure 5). High astaxanthin extractability was obtained at temperatures lower than 35 °C and a pH of approximately 4.5. According to the model, the highest astaxanthin extraction could be obtained at a temperature of 30.8 °C and at pH 4.6. This set of values predicted an astaxanthin extractability of 94% (dry cells) and 116% (wet cells), respectively. The models were then validated by repeating the experiments using the critical parameters (temperature at 30.8 °C and pH 4.6) and revealed a satisfactory prediction, with 105% (*w/w*) astaxanthin extraction for dry and 180% (*w/w*) for wet cells.

**Figure 6:**
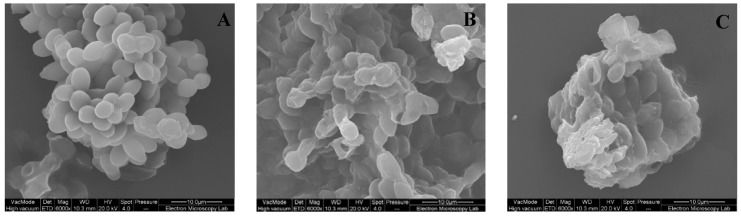
SEM images depicting *X. dendrorhous* (**A**) intact cells, (**B**) after Accelerace 1500 pre-treatment, and (**C**) after Glucanex pre-treatment.

This study demonstrated that astaxanthin extractability using Glucanex was considerably higher than using Accellerase 1500. This probably occurred due to the differences in the activities of these two commercial formulations, which included β-glucanase, cellulase, protease, and chitinase; as demonstrated experimentally, the β-glucanase activity of Glucanex was significantly higher than that of Accellerase 1500. The high β-glucanase activity most likely led to cell lysis through the hydrolysis of the structure of (1–3)-glucose of the yeast cell wall glucans. As a result, the hydrolysed cell walls allowed acetone to permeate into the cells and extract astaxanthin. The high astaxanthin extractability values obtained when using Glucanex was supported by the SEM images before and after extraction (Figure 6). The images show that after Glucanex treatment, the cells had irregular shapes and were shrunk in size (Figure 6C) compared to fresh cells (Figure 6A); in the case of Accellerace 1500, a portion of the cells still maintained their cell integrity (Figure 6B).

Enzymatic cell lysis in yeast occurs as a synergistic effect of protease and glucanase activities [41]. Generally, enzymatic cell lysis in yeast starts with the binding of the lytic protease to the outer mannoprotein layer of the cell wall, which results in exposure of the protein structure and releases the cell wall protein and mannan while exposing the glucan surface. Subsequently, the glucanase enzyme attacks the inner cell wall and solubilises the glucans [26]. The soluble structure of yeast allows then acetone to permeate through the cell wall and solubilise astaxanthin. Enzymatic cell lysis as the means for extracting products of metabolism from yeast cells has gained wide interest among researchers and has been used to produce yeast extract from *Saccharomyces cerevisiae* [25]. In terms of astaxanthin extraction from yeast cells, Michelon et al. [41] combined maceration with diatomaceous earth and Glucanex lysis, and this process resulted in 122% extraction yield of carotenoids from *Phaffia rhodozyma*, compared to DMSO extraction. In another study, enzymatic cell lysis using different types of enzymes (Glucanex, Lyticase, and Driselase) was combined with ultrasound pre-treatment to extract astaxanthin inform the microalgae *Haematococcus pluvialis* [42]. This strategy resulted in 84% of astaxanthin extraction compared to standard chemical extraction methods. Overall, these studies indicate that there is considerable potential in using enzymatic cell lysis with other cell disruption methods to increase astaxanthin extractability from yeast cells.

### 3.4. Supercritical Fluid Extraction as Means of Astaxanthin Extraction

Supercritical CO_2_ was investigated as an alternative solvent for astaxanthin extraction from *X. dendrorhous* cells, by employing also ethanol as a co-solvent. Yeast cells pre-treated with Accellerase 1500 were used as extraction material; the particular enzyme was found to moderately disrupt the yeast cell wall; therefore, an additional objective was to investigate whether the combination of the two treatments would increase astaxanthin extractability overall. A non-factorial central composite design of experiments (DoE), which included twelve factorial runs and five zero-point runs, was conducted (Table 3). Both pressure and co-solvent concentrations significantly influenced astaxanthin extraction. Co-solvent (ethanol) acts by increasing the polarity of CO_2_, allowing the dissolution of polar compounds. Even though astaxanthin has low polarity, it has a high molecular weight (MW = 596.8 g/mol). Therefore, the presence of ethanol facilitates the extraction process as it can aid the dissolution of heavier substances in CO_2_ [43].

It was observed that increasing the ethanol concentration presented a positive correlation with astaxanthin extractability. This was observed in Runs 3 and 7, as an increase in EtOH from 5% to 15% resulted in increased astaxanthin extractability, from 16.4% to 21.7%. In terms of the influence of pressure, a similar trend was observed, as an increase in pressure positively affected astaxanthin extractability. This was observed in Runs 11 and 12, where an increase of pressure from 82 to 418 bar resulted in a significant increase in astaxanthin extractability (from ~0% to ~13%). The higher pressure most likely led to a greater disruption of the yeast cell wall and caused the release of the pigment, similar to what has been previously shown for plant cell wall structures [43].

A model that predicts the influence of temperature, pressure, and ethanol concentration on astaxanthin extraction by supercritical CO2 was generated and the equation of the quadratic model is given below (Equation (7)). It was observed that only the linear term of pressure and ethanol concentration demonstrated a statistically significant (*p* < 0.05) effect. The linear term of temperature, the quadratic term of temperature, pressure, and ethanol concentration, as well as the interaction terms between these variables did not have a significant effect (*p* > 0.05).

(7) $$Astaxanthin\;Extractability\;\left(\%\right)=18.5-0.832T+0.0379P+1.13E+0.00434\;T^{2}-0.000114P^{2}-0.0011E^{2}+0.001121TP+0.0001TE-0.00227PE$$

where *T* = temperature, *p* = pressure, and *E* = ethanol.

An ANOVA test was performed to confirm the accuracy of the model, along with an F-test. The coefficient of regression (R^2^) was 0.8846 and the F-value (5.96) was higher than the tabulated F-value (F_9,7_ = 2.72), indicating that the model gave a good fit and was able to describe satisfactorily the astaxanthin extraction process from yeast cells using supercritical CO_2_. According to the model the maximum predicted maximum astaxanthin extractability (25.7%) was predicted at 76.8 °C, 360 bar, and 18% (*v/v*) ethanol concentration. The model was then validated by repeating the experiments using these critical parameters and an astaxanthin extraction yield of 22% (*w/w*) was obtained, demonstrating a satisfactory prediction of the extraction process by the model.

The results obtained in this study are in line with previous works in this area, concerning the extraction of pigments and lipids. Previous research incorporated a cell pre-treatment step using a bead mill to disrupt the *Phaffia rhodozyma* cells prior to supercritical CO_2_ extraction [44]. This strategy was found to increase astaxanthin extractability ~90% under optimised conditions (temperature = 40 °C, pressure = 500 bar). In another study, Duarte et al. [45] investigated the use of supercritical CO_2_ in combination with ultrasound treatment, to extract intracellular lipids from the yeast *Candida* sp. LEB-M3. They found that pre-treatment with ultrasonication followed by CO_2_-SCFE resulted in a relatively low lipid extractability (20%) as compared to the conventional chemical extraction method, indicating that the ultrasonication method was not able promote significant cell rupture. Besides that, the effect of the pre-treatment step and the type of microorganism is likely to affect considerably the performance of the supercritical CO_2_ process. For example, a supercritical CO_2_ method was used to extract carotenoids from microalgae species, in particular *Haematococcus pluvialis*. Under optimised conditions (temperature = 55 °C, pressure = 20 MPa, and EtOH = 13%), 84% of astaxanthin extractability was obtained using supercritical CO_2_, using disrupted, freeze-dried cells [23]. Overall, the present work as well as previous works demonstrate that there is significant scope for further research combining different pre-treatment methods (i.e., enzymatic, chemical, and physical) with supercritical CO_2_ to maximise the extraction of astaxanthin from yeast cells.

## 4. Conclusions

This study allowed an assessment of a number of strategies for the extraction of astaxanthin from *X. dendrorhous* cells. The best extraction strategy involved the use of Glucanex (5% *w/v*) followed by acetone extraction; this resulted in even higher astaxanthin extraction compared to the standard extraction method (DMSO/acetone). The use of Accellerase (30% *v/v*) to disrupt the cell wall did not help in extracting astaxanthin (using acetone as the solvent) mainly due to the different enzyme activities present in the two enzymes, and particularly the low β-1, 3 glucanase activity of Accellerase 1500. Supercritical CO_2_ showed potential although an enzymatic pre-treatment step was deemed necessary to rupture the cell wall structure and reach extractability values of ~45%. Nevertheless, astaxanthin extraction by supercritical CO_2_ still holds the potential for large-scale operations, taking into account its potential environmental advantages (less solvent).

## Figures and Tables

**Table 1:** Effect of pH and temperature on astaxanthin extractability (%, *w/w*) of dry and wet samples of *X. dendrorhous* DSMZ 5627 cells after treatment with Accellerase 1500.

Run	pH	Temperature (°C)	Astaxanthin Extractability (%, *w/w*)	
			Dry Cells	Wet Cells
**1**	4.50 (−1)	30.0 (−1)	6.33	10.59
**2**	6.50 (+1)	30.0 (−1)	4.91	6.98
**3**	4.50 (−1)	50.0 (+1)	4.84	8.85
**4**	6.50 (+1)	50.0 (+1)	4.78	5.30
**5**	4.09 (−1)	40.0 (0)	8.66	14.08
**6**	6.91 (+1)	40.0 (0)	4.65	5.81
**7**	5.50 (0)	25.7 (−1)	4.97	9.30
**8**	5.50 (0)	54.1 (+1)	2.52	4.00
**9**	5.50 (0)	40.0 (0)	8.91	12.60
**10**	5.50 (0)	40.0 (0)	9.12	13.50
**11**	5.50 (0)	40.0 (0)	7.49	13.11
**12**	5.50 (0)	40.0 (0)	7.95	12.95
**13**	5.50 (0)	40.0 (0)	9.43	13.76

**Table 2:** Effect of pH and temperature on astaxanthin extractability (%, *w/w*) of dry and wet samples of *X. dendrorhous* DSMZ 5627 cells after treatment with Glucanex.

Run	pH	Temperature (°C)	Astaxanthin Extractability (%, *w/w*)	
			Wet Cells	Dry Cells
**1**	3.5 (−1)	35.0 (−1)	91.80	79.80
**2**	3.5 (−1)	55.0 (+1)	39.95	22.04
**3**	5.50 (+1)	35.0 (−1)	93.33	76.91
**4**	5.50 (+1)	55.0 (+1)	43.00	18.09
**5**	4.5 (0)	30.9 (−1)	114.83	84.36
**6**	4.5 (0)	59.1 (+1)	47.58	25.99
**7**	3.09 (−1)	45.0 (0)	44.83	22.04
**8**	5.91 (+1)	45.0 (0)	60.24	35.57
**9**	4.50 (0)	45.0 (0)	88.91	61.41
**10**	4.50 (0)	45.0 (0)	90.89	66.73
**11**	4.50 (0)	45.0 (0)	95.16	63.23
**12**	4.50 (0)	45.0 (0)	84.94	61.26
**13**	4.50 (0)	45.0 (0)	90.89	65.36

**Table 3:** Effect of temperature, pressure, and co-solvent concentration (ethanol) on astaxanthin extractability (%, *w/w*) of *X. dendrorhous* Accellerase-pre-treated cells.

Run	Temperature (°C)	Pressure (bar)	EtOH (%)	Astaxanthin Extraction (%, *w/w*)
**01**	50.0 (−1)	150.0 (−1)	5.0 (−1)	7.78
**02**	70.0 (+1)	150.0 (−1)	5.0 (−1)	1.62
**03**	50.0 (−1)	350.0 (+1)	5.0 (−1)	16.37
**04**	70.0 (+1)	350.0 (+1)	5.0 (−1)	17.94
**05**	50.0 (−1)	150.0 (−1)	15.0 (+1)	20.33
**06**	70.0 (+1)	150.0 (−1)	15.0 (+1)	14.50
**07**	50.0 (−1)	350.0 (+1)	15.0 (+1)	21.70
**08**	70.0 (+1)	350.0 (+1)	15.0 (+1)	23.01
**09**	43.2 (−1)	250.0 (0)	10.0 (0)	13.40
**10**	76.8 (+1)	250.0 (0)	10.0 (0)	14.82
**11**	60.0 (0)	81.8 (−1)	10.0 (0)	0.04
**12**	60.0 (0)	418.2 (+1)	10.0 (0)	13.38
**13**	60.0 (0)	250.0 (0)	1.6 (0)	4.24
**14**	60.0 (0)	250.0 (0)	18.4 (+1)	19.64
**15**	60.0 (0)	250.0 (0)	10.0 (0)	14.30
**16**	60.0 (0)	250.0 (0)	10.0 (0)	14.32
**17**	60.0 (0)	250.0 (0)	10.0 (0)	16.52
